# Unexpected intrathecal haemorrhage following uncomplicated placement and removal of an epidural catheter

**DOI:** 10.1259/bjrcr.20170108

**Published:** 2018-06-05

**Authors:** Vivek Singh, Sumir Patel, Kush Singh

**Affiliations:** 1 Virginia Tech Carilion School of Medicine, Roanoke, Virginia, VA, USA; 2 Department of Radiology, Emory University Hospital, Atlanta, Georgia, GA, USA

## Abstract

We report a case of intrathecal and epidural haemorrhage 2 weeks after uncomplicated placement and removal of an epidural catheter in a patient that was initially scheduled for surgical repair of an aortic dissection and aneurysm. Included in this case report is a literature review and discussion of similar entities, differential diagnoses, and high yield learning points.

## Case

A 59-year-old African American male with known chronic Stanford type B aortic dissection with extension into a thoracoabdominal aneurysm was admitted for elective repair. Pre-operatively, an epidural catheter was placed by and for anaesthesia on the first attempt without any complication. Upon further investigation, patient admitted to continued tobacco use leading up to the surgery and demonstrated poor performance on pre-operative pulmonary function tests. In light of these findings, his surgery was delayed until a later time, the epidural catheter was removed 3–4 h after initial placement, and the patient was discharged with follow up within 1 month. The patient reported no immediate symptoms after catheter removal.

11 days later, the patient presented to the emergency department with a 5-day history of constant, global, 10/10 headache that was significantly worse when standing. He also noted accompanying severe, constant lumbar spine pain with bilateral lower extremity weakness but denied numbness or tingling. Additional pertinent medical history included coronary artery disease status post-bare metal stent, chronic obstructive pulmonary disease, Stage II chronic kidney disease, hypertension, and ongoing tobacco use.

On physical examination, the patient was alert, oriented, and demonstrated no focal neurological deficits, but did display tenderness to palpation over the lumbar spine at the site where the epidural catheter had been placed and subsequently removed.

**Figure 1.**
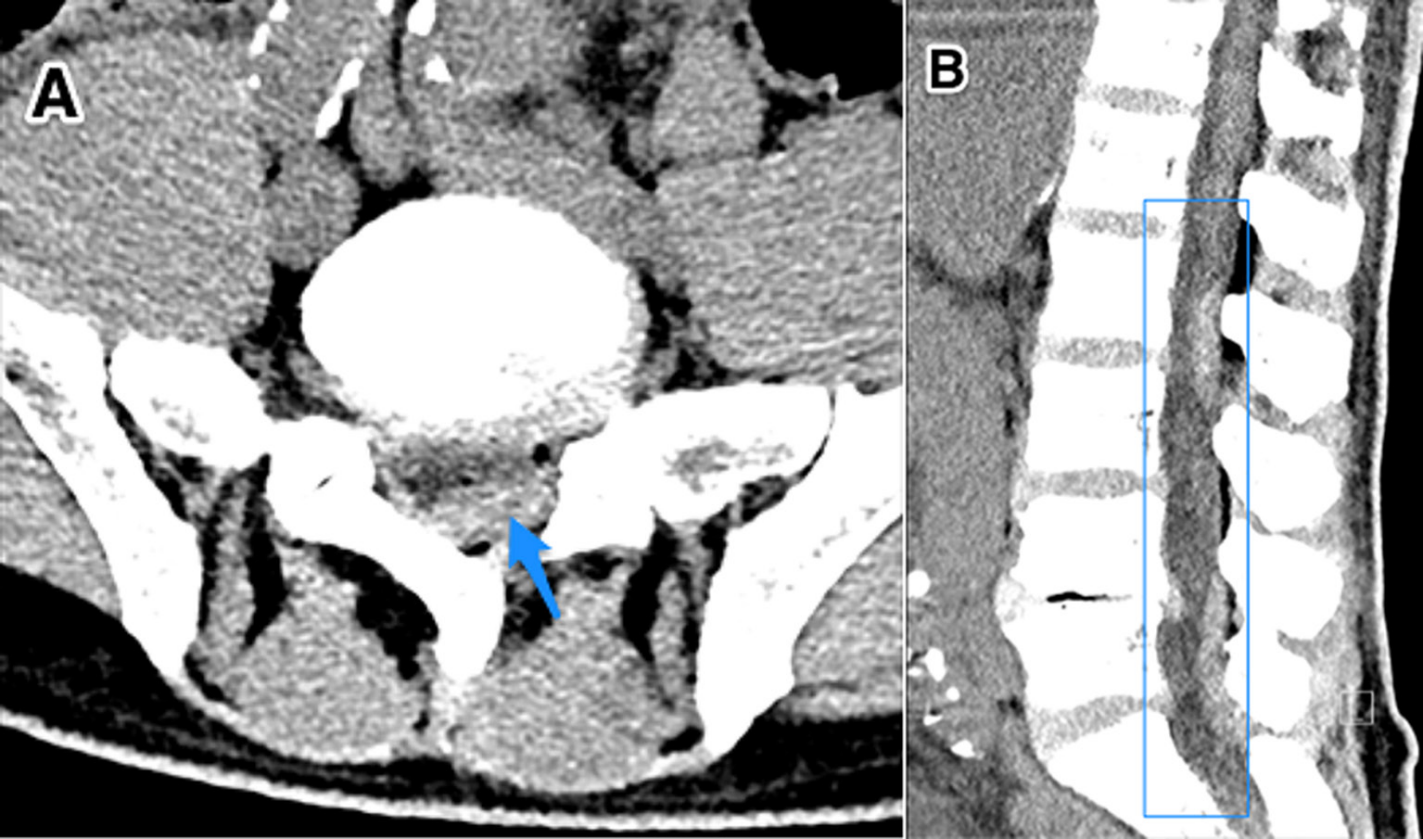
A 59-year-old male with intrathecal haemorrhage at the mid-L2 to upper third L3 level secondary to epidural catheter placement. Findings: axial (a) and sagittal (b) CT of the lumbar spine demonstrating a layering hyperdensity within the lumbar thecal sac extending from the mid-L2 level to mid-L3 level with a second collection layering dependently from L4-L5 and L5-S1. Technique: standard CT of the lumbar spine with contrast.

CT of the head without contrast performed in the Emergency Department was unremarkable. CT of the lumbar spine without contrast showed layering hyperdensity extending from the mid L2 vertebral body to the mid L3 level as well as a second hyperdense collection layering dependently from L4-S1 (Figure 1).

**Figure 2.**
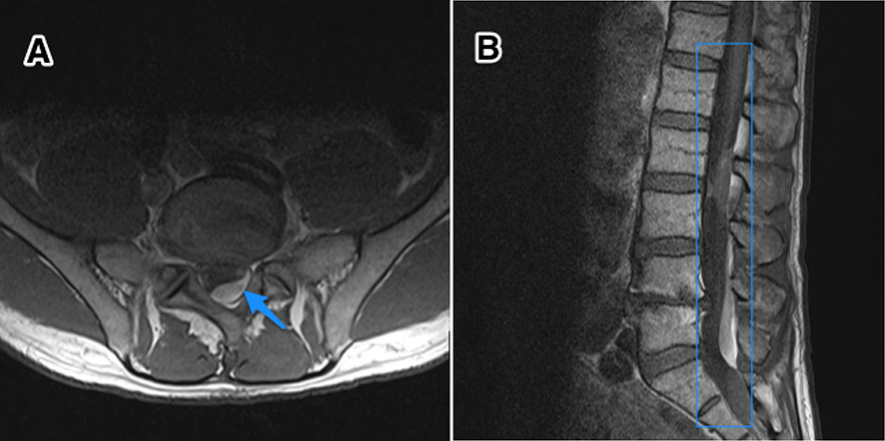
A 59-year-old male with intrathecal haemorrhage at the mid-L2 to upper third L3 level secondary to epidural catheter placement. Findings: axial (Figure 1a) and sagittal (b) *T*_1_ weighted images of the lumbar spine showing intrathecal blood products of varying ages. *T*_1_ hyperintense blood products are also seen extending from L4 through S1. Technique: sagittal *T*_1_ weighted with i.v. contrast 1.5 T-MRI of the lumbar spine.

MRI of the lumbar spine later that day redemonstrated a stable intrathecal haemorrhage and an epidural haematoma with anteromedial displacement of the cauda equina, most notably from the mid L2 to upper L3 vertebral bodies (Figure 2).

## DISCUSSION

### Aetiology & demographics

Intrathecal bleeding can be subdural, subarachnoid, or intramedullary based on location with respect to the thecal sac. Due to its extremely low incidence, no specific guidelines regarding diagnosis or management exist.^1^ Haematoma formation within the thecal sac is rare because normal clotting mechanism is hindered by dilution of blood by cerebrospinal fluid and defibrination by normal pulsation.^2^ The most frequent causes of intrathecal bleeding include coagulopathies (40.5%), lumbar puncture for diagnostic or anaesthesiological purposes (44.9%), and traumatic injuries (15.9%), although the various individual aetiologies may be combined.^3^ The theorized pathogenesis of iatrogenic intrathecal haemorrhage involves rupture of the radicular arteries and veins. Others have suggested that minor trauma coupled with changes in intrathoracic and intra-abdominal pressure yield an increased luminal pressure within vessels of the subarachnoid space, causing rupture of these vessels.^4^ Literature review of similar cases between 1974 and 2014 shows an even distribution among males (51%) and females (49%). Ages in these cases ranged from 17 months to 83 years with a mean of 48 years, indicating a symmetric distribution. Of these patients, 42.9% had some underlying coagulopathy (Table 1) .^5^

**Table 1.** Summary table of intrathecal haemorrhage

Aetiology	Iatrogenic
Incidence	Unknown
Gender ratio	1:1^5^
Age predilection	Median age of 48 with symmetric distribution^5^
Risk factors	Pre-existing coagulopathy, traumatic tap, intracranial subarachnoid haemorrhage^5^
Treatment	Surgical decompression in the presence of neurological deficits; otherwise conservative medical management^3^
Prognosis	No significant difference between surgical *v* *s* non-surgical intervention^5^
Imaging findings	CT—hyperdensity within the lumbar thecal sac MRI—intermediate *T*_1_ and *T*_2_ signal material within thecal sac

### Clinical & imaging findings

Clinical manifestation of intrathecal haemorrhage is usually delayed 2–4 days after the trigger event. Symptoms may include sudden back pain or headache, acute sciatic pain, lower extremity weakness, paraparesis, sensory changes, and/or sphincter disturbance.^6^ Clinical picture is significantly influenced by both composition and location of the bleed. Komiyama, et al suggest that haemorrhages more ventral in position within the thecal sac typically present with isolated acute back pain, whereas haemorrhage more dorsal in position is more likely to cause displacement of the cauda equina, increasing the likelihood of significant neurological deficits.^1, 7^

The gold-standard for diagnosis of intrathecal haemorrhage is MRI, which should be done rapidly for early diagnosis and management. CT imaging, with or without myelography, can also be used but its specificity is limited by diminished image resolution.^8^

Differentiation between epidural, subdural, and subarachnoid bleeds can be particularly challenging as there may be mixed presentation at contiguous levels (Table 2).^9^ Epidural haematomas are associated with a convex appearance, are located dorsally, and cause ventral dural displacement. If located ventrally, the haematoma can display a “curtain sign” from attachment of dura to the posterior longitudinal ligament by Hoffman’s ligament.^10^ Subdural haematomas are associated with a crescent shape on axial images, resulting in a semi-circular appearance. It is also commonly recognized as an inverted “Mercedes-Benz sign”.^11^ Differentiation can also be assisted by the presence of a black line on gradient echo *T*_2_ weighted MRI, which represents an oedematous arachnoid between abnormal signals and the cauda equina.^9^ Finally, in the case of subarachnoid haemorrhage, blood will have a heterogeneous appearance on MRI secondary to dilution with cerebrospinal fluid, defibrination from normal pulsations, and the presence of intermixed nerve roots.^12^ Acutely, SAH will appear hyper- or isointense on *T*_1_- and hyper- or hypointense on *T*_2_ MRI.^13^ Subacute haemorrhage becomes hyper- or isointense on *T*_1_, while *T*_2_ displays a hyperintense signal due to the strongly paramagnetic methaemoglobin.^9^

**Table 2.** Differential diagnosis table for intrathecal haemorrhage

Differential Diagnosis	CT	MRI
Subarachnoid haemorrhage	Heterogeneous hyperdensity within the subarachnoid space^9^	*T*_1_ hyper- or isointense signal, *T*_2_ hyper- or hypointense signal within the subarachnoid space^9^
Epidural haematoma	Convex shaped hyperdensity located dorsally with ventral displacement of dura^10^	Dependent on blood age; in acute, extradural *T*_1_ iso- or hyperintense to spinal cord; *T*_2_ heterogeneously hyper- to spinal cord with hypointense foci^10^
Subdural haematoma	Crescent shaped hyperdensity on axial images, associated with “inverted Mercedes-Benz sign.”^11^	Dependent on blood age; in acute, *T*_1_ iso or hyperintense to spinal cord; *T*_2_ heterogeneously hyperintense to spinal cord with hypointense foci within the dura^11^

CAD, coronary artery disease; COPD, chronic obstructive pulmonary disease; CKD, chronic kidney disease;  HTN, hypertension; PFT, pulmonary function test; SAH, subarachnoid haemorrhage.

### Treatment & prognosis

Management of intrathecal haemorrhage is dictated by the neurological status of the patient. As mentioned previously, the presence or absence of neurological deficits relates to both the location and composition of the bleed. Most case reports suggest surgical evacuation of haematoma or haemorrhage in the presence of neurological deficit or deterioration. Alternative treatments include needle aspiration and conservative medical management.^1, 7,12^ The overall mortality of intrathecal haemorrhage is 25.7%.^3^ Prognosis is largely dependent on pre-operative neurological function, duration between onset of symptoms and surgery, and rapidity of deterioration.^14^ Recovery of neurological function is more favourable if surgery occurs within 8 h of onset of symptoms. Additionally, patients with lumbosacral haemorrhages (L2-S1) had improved recovery compared with those patients with haemorrhages that compressed the spinal cord at more superior levels (C1-L1).^15^ Furthermore, patients who had some underlying coagulopathy had statistically significantly poorer outcomes (28.6%) than non-coagulopathic patients (14.3%). Overall comparison of surgical *v* *s* non-surgical intervention revealed no significant difference in outcomes.^5^

### Differential diagnosis

The differential diagnosis of intrathecal haemorrhage is largely based on other pathologies involving anatomically adjacent structures. Clinical differentiation between epidural, subdural, and subarachnoid bleeds or haematomas can be difficult, as all these entities can result in very similar presentation with localized pain and possible neurological deficits secondary to compression of nerve roots in the area. The presence of intractable headache accompanied by severe back pain raises the index of suspicion for an intracranial subarachnoid haemorrhage, which may have triggered the spinal bleed. The differential diagnosis for the aetiology of an intrathecal bleed is broad and includes bleeding disorders, coagulopathy, thromboprophylaxis, autoimmune disease (*i.e*. Behcet Syndrome), trauma, neoplasia, and arteriovenous malformation.^1, 3,4^

### Case discussion

In our case, there were several factors that may have contributed to the intrathecal haemorrhage. First, the patient was thrombocytopaenic at the time of epidural catheter placement with a platelet count of 138,000 μl^–1^. Additionally, the patient had a known history of aortic dissection with thoracoabdominal extension into an aneurysm with accompanying hypertension, increasing his risk of bleeding. However, contrary to these factors, his laboratory results showed he had a slightly low activated partial thromboplastin time of 24.2, a normal prothrombin time of 12.2, and a normal international normalized ratio of 1.06. Furthermore, it should be noted that his blood pressure at the time of the epidural catheter placement was relatively well controlled (133/77). Also, the epidural catheter was both placed and removed easily with single attempts with minimal bleeding. The patient denied any paresthesia, weakness, or pain during or immediately after the procedure. Although he had a history of coronary artery disease, he was not on any anticoagulation at the time, which should have significantly decreased his risk for haemorrhage following a non-traumatic lumbar puncture.

Given the MRI findings of an intrathecal haemorrhage with epidural haematoma and lack of neurological deficits, the patient was managed conservatively with blood pressure and pain control. He was discharged from the hospital with follow up scheduled at a later time to ensure resolution of the haemorrhage and eventual repair of his dissection/aneurysm.

## Learning POINT

1. Intrathecal haemorrhage can occur secondary to non-traumatic lumbar puncture or even epidural catheter placement in patients who are not anticoagulated. Clinicians should be suspicious of this entity in patients who present with severe back pain and neurological deficits following remote history of spinal trauma, as in the case of this patient with prior epidural catheter placement. Additionally, radiologists should be aware that intrathecal haemorrhage can remain in the thecal sac for weeks after the initial insult as was seen in this patient 2 weeks after catheter placement and immediate removal.
2. Early MRI can offer diagnostic clarity, as this modality is both sensitive and specific for this entity. Urgent surgical evacuation is indicated in the presence of neurological deficits or rapid deterioration.
3. Management is dictated by the neurological status of the patient. There is no significant difference between surgical vs non-surgical intervention although surgical evacuation of the hematoma within 8 h results in more favourable outcomes.

## References

[1] Walsh KM, Vedant V, Schlenk RP Transient paraparesis from a traumatic lumbar intratehcal hemorrhage. A case report and literature review. J Neurol Disord 2016; 4: 254.

[2] Rengachary SS, Murphy D Subarachnoid hematoma following lumbar puncture causing compression of the cauda equina. Case report. J Neurosurg 1974; 41: 252–4. doi: 10.3171/jns.1974.41.2.0252 4841881

[3] Domenicucci M, Ramieri A, Paolini S, Russo N, Occhiogrosso G, Di Biasi C, et al Spinal subarachnoid hematomas: our experience and literature review. Acta Neurochir 2005; 147: 741–50. doi: 10.1007/s00701-004-0458-2 15711890

[4] Moore JM, Jithoo R, Hwang P Idiopathic spinal subarachnoid hemorrhage: a case report and review of the literature. Global Spine J 2015; 5: 59–64. doi: 10.1055/s-0035-1546416 26430603 PMC4577320

[5] Brown MW, Yilmaz TS, Kasper EM Iatrogenic spinal hematoma as a complication of lumbar puncture: What is the risk and best management plan? Surg Neurol Int 2016; 7(Suppl 22): S581. doi: 10.4103/2152-7806.189441 27625895 PMC5009572

[6] Avecillas-Chasín JM, Matias-Guiu JA, Gomez G, Saceda-Gutierrez J A case of acute spinal intradural hematoma due to spinal anesthesia. JAD 2015; 4: 341–3. doi: 10.1016/j.joad.2015.06.015

[7] Komiyama M, Yasui T, Sumimoto T, Fu Y Spontaneous spinal subarachnoid hematoma of unknown pathogenesis: case reports. Neurosurery 41: 693–4. 10.1097/00006123-199709000-00040 9310992

[8] Shimada Y, Sato K, Abe E, Miyakoshi N, Tsutsumi Y Spinal subdural hematoma. Skeletal Radiol 1996; 25: 477–80. doi: 10.1007/s002560050118 8837281

[9] Kakitsubata Y, Theodorou SJ, Theodorou DJ, Miyata Y, Ito Y, Yuki Y, et al Spontaneous spinal subarachnoid hemorrhage associated with subdural hematoma at different spinal levels. Emerg Radiol 2010; 17: 69–72. doi: 10.1007/s10140-008-0792-4 19184145 PMC2773034

[10] Küker W, Thiex R, Friese S, Freudenstein D, Reinges MH, Ernemann U, et al Spinal subdural and epidural haematomas: diagnostic and therapeutic aspects in acute and subacute cases. Acta Neurochir 2000; 142: 777–85. 10955672 10.1007/s007010070092

[11] Johnson PJ, Hahn F, McConnell J, Graham EG, Leibrock LG The importance of MRI findings for the diagnosis of nontraumatic lumbar subacute subdural haematomas. Acta Neurochir 1991; 113: 186–8. doi: 10.1007/BF01403207 1799164

[12] Ruelle A, Zerbi D, Andrioli G Spinal subarachnoid bleeding of unknown etiology. Case reports. J Neurosurg Sci 2001; 45: 53–7. 11466509

[13] Kim YH, Cho KT, Chung CK, Kim HJ Idiopathic spontaneous spinal subarachnoid hemorrhage. Spinal Cord 2004; 42: 545–7. doi: 10.1038/sj.sc.3101620 15111997

[14] Sunada I, Akano Y, Kidosaki Y, Shimokawa N, Yamamoto S Spontaneous spinal subarachnoid hematoma-case report. Surg Neurol 1995; 44: 133–6. doi: 10.1016/0090-3019(95)00166-2 7502202

[15] Vandermeulen EP, Van Aken H, Vermylen J Anticoagulants and spinal-epidural anesthesia. Anesth Analg 1994; 79: 1165–77. doi: 10.1213/00000539-199412000-00024 7978443

